# Challenges and Opportunities With Routinely Collected Data on the Utilization of Cancer Medicines. Perspectives From Health Authority Personnel Across 18 European Countries

**DOI:** 10.3389/fphar.2022.873556

**Published:** 2022-06-16

**Authors:** Alice Pisana, Björn Wettermark, Amanj Kurdi, Biljana Tubic, Caridad Pontes, Corinne Zara, Eric Van Ganse, Guenka Petrova, Ileana Mardare, Jurij Fürst, Marta Roig-Izquierdo, Oyvind Melien, Patricia Vella Bonanno, Rita Banzi, Vanda Marković-Peković, Zornitsa Mitkova, Brian Godman

**Affiliations:** ^1^ Department of Global Public Health, Karolinska Institutet, Stockholm, Sweden; ^2^ Department of Pharmacy, Faculty of Pharmacy, Disciplinary Domain of Medicine and Pharmacy, Uppsala University, Uppsala, Sweden; ^3^ Faculty of Medicine, Vilnius University, Vilnius, Lithuania; ^4^ Strathclyde Institute of Pharmacy and Biomedical Science, University of Strathclyde, Glasgow, United Kingdom; ^5^ Department of Pharmacology and Toxicology, College of Pharmacy, Hawler Medical University, Erbil, Iraq; ^6^ Division of Public Health Pharmacy and Management, School of Pharmacy, Sefako Makgatho Health Sciences University, Pretoria, South Africa; ^7^ Department of Pharmacy, Faculty of Medicine, University of Banja Luka, Banja Luka, Bosnia and Herzegovina; ^8^ Catalan Health Service, Barcelona, Spain; ^9^ Department of Pharmacology, Therapeutics and Toxicology, Universitat Autònoma de Barcelona, Barcelona, Spain; ^10^ RESHAPE, INSERM U1290 & Claude Bernard University Lyon 1, Lyon, France; ^11^ Asthma Self Care Training Unit, Respiratory Medicine, Croix Rousse University Hospital, Lyon, France; ^12^ PELyon, PharmacoEpidemiology Lyon, Lyon, France; ^13^ Public Health and Management Department, Faculty of Medicine, “Carol Davila” University of Medicine and Pharmacy, Bucharest, Romania; ^14^ Health Insurance Institute, Ljubljana, Slovenia; ^15^ Head of Section for Drug Therapeutics and Safety, Oslo University Hospital, Oslo, Norway; ^16^ Head of National Center for Drug Shortage in Specialist Health Care, Department of Pharmacology, Oslo University Hospital, Oslo, Norway; ^17^ Division of Pharmacoepidemiology, Strathclyde Institute of Pharmacy and Biomedical Sciences, University of Strathclyde, Glasgow, United Kingdom; ^18^ Department of Health Systems Management and Leadership, Faculty of Health Sciences, University of Malta, Msida, Malta; ^19^ Center for Health Regulatory Policies, Istituto di Ricerche Farmacologiche Mario Negri IRCCS, Milan, Italy; ^20^ Department of Social Pharmacy, Faculty of Medicine, University of Banja Luka, Banja Luka, Bosnia and Herzegovina; ^21^ Centre of Medical and Bio-allied Health Sciences Research, Ajman University, Ajman, United Arab Emirates

**Keywords:** new cancer medicines, patient-level datasets, pricing and reimbursement, funding concerns, pharmaceutical policy, cross-national collaboration, european countries

## Abstract

**Background:** Rising expenditure for new cancer medicines is accelerating concerns that their costs will become unsustainable for universal healthcare access. Moreover, early market access of new oncology medicines lacking appropriate clinical evaluation generates uncertainty over their cost-effectiveness and increases expenditure for unknown health gain. Patient-level data can complement clinical trials and generate better evidence on the effectiveness, safety and outcomes of these new medicines in routine care. This can support policy decisions including funding. Consequently, there is a need for improving datasets for establishing real-world outcomes of newly launched oncology medicines.

**Aim:** To outline the types of available datasets for collecting patient-level data for oncology among different European countries. Additionally, to highlight concerns regarding the use and availability of such data from a health authority perspective as well as possibilities for cross-national collaboration to improve data collection and inform decision-making.

**Methods:** A mixed methods approach was undertaken through a cross-sectional questionnaire followed-up by a focus group discussion. Participants were selected by purposive sampling to represent stakeholders across different European countries and healthcare settings. Descriptive statistics were used to analyze quantifiable questions, whilst content analysis was employed for open-ended questions.

**Results:** 25 respondents across 18 European countries provided their insights on the types of datasets collecting oncology data, including hospital records, cancer, prescription and medicine registers. The most available is expenditure data whilst data concerning effectiveness, safety and outcomes is less available, and there are concerns with data validity. A major constraint to data collection is the lack of comprehensive registries and limited data on effectiveness, safety and outcomes of new medicines. Data ownership limits data accessibility as well as possibilities for linkage, and data collection is time-consuming, necessitating dedicated staff and better systems to facilitate the process. Cross-national collaboration is challenging but the engagement of multiple stakeholders is a key step to reach common goals through research.

**Conclusion:** This study acts as a starting point for future research on patient-level databases for oncology across Europe. Future recommendations will require continued engagement in research, building on current initiatives and involving multiple stakeholders to establish guidelines and commitments for transparency and data sharing.

## 1 Introduction

*Cancer* is a major global health challenge, with currently almost 10 million deaths annually and an estimated 19.3 million new cases occurring in 2020 (Sung et al., 2021). This burden is consistently growing, with a projected rise to 28.4 million new cancer cases globally in 2040 (Sung et al., 2021). *Cancer* also has a high and growing economic burden, with an estimated US$1.16 trillion spent on direct costs in 2010 and rising (McCormick, 2018). In Europe, between 1995 and 2018 direct costs due to cancer increased by 98% from €52 billion to €103 billion, constituting 6.2% of total health expenditure in 2018 (Hofmarcher et al., 2019). Much of this increase was attributed to higher expenditure for cancer medicines (Hofmarcher et al., 2019). Overall, expenditure on oncology medicines in Europe increased from €12.9 billion to €32.0 billion between 2009 and 2018 (Hofmarcher et al., 2019) and is expected to rise further. This is attributed to the increasing prevalence of cancer, as well as the development and early launch of new high-priced treatments, with over 500 companies currently investing in new cancer medicines for more than 600 indications (Pontes et al., 2020; Godman et al., 2021a), exacerbated by the emotive nature of the disease (Haycox, 2016; Cohen, 2017). New cancer medicines continue to dominate research and development activities among pharmaceutical companies (IQVIA, 2022).

This issue of affordability of new cancer medicines is an increasing concern among European and other countries (Hirsch et al., 2014; Vogler et al., 2016; Godman et al., 2017; Vogler et al., 2017; Godman et al., 2021a; Vogler, 2021), with the cost of cancer care accounting for up to 30% of total hospital expenditure across Europe and rising (Simoens et al., 2017). There are similar concerns in the US where expenditures on new oncology medicines approved in 2018 alone could be as high as US$39.5 billion per year if prescribed to all eligible patients (Demartino et al., 2020). Furthermore, there is a constant pressure to quickly fund and facilitate market access to new oncology treatments, even with only limited clinical trial data, in order to try and address continued unmet medical need (Pontes et al., 2020). Consequently, current funding and reimbursement models especially for new cancer medicines often place a heavy strain on healthcare systems and will impact on the sustainability of universal healthcare in Europe (Godman et al., 2021a; Vogler, 2021). This has resulted in the development of new pricing models including managed entry agreements (MEAs) and multiple criteria decision analysis as well as better systems for the introduction and follow-up of new medicines including horizon scanning and budget-forecast activities (Godman et al., 2014a; Godman et al., 2021a).

Various regulatory mechanisms have also been introduced including adaptive licensing (Eichler et al., 2012; Eichler et al., 2015; Vella Bonanno et al., 2017), accelerated assessments and conditional marketing approval, to facilitate authorization and funding of promising candidate medicines early in their development (Hoekman et al., 2015; Martinalbo et al., 2016). However, there are concerns with such proposals due to the lack of robust evidence for improved outcomes of these new medicines when used in routine clinical practice (Godman et al., 2014a; Beachler et al., 2021). In addition, currently new oncology treatments are often evaluated based on Phase II and III trials using surrogate endpoints, which are easier to measure (Kim and Prasad, 2015; Kemp and Prasad, 2017). For instance, in the US in 2017, 21% of new medicines for patients with cancer were approved by the US Food and Drug Administration (FDA) based on Phase I/II trials with 50% based on Phase II trials (IQVIA Institute for Human Data Science, 2018). This is a concern for health authorities, as surrogate markers do not necessarily translate into improved survival rates in practice, leaving considerable uncertainty in terms of the overall clinical benefit and therapeutic value of new medicines (Cortazar et al., 2014; Prasad et al., 2015; Kemp and Prasad, 2017; Paoletti et al., 2020). Uncertainty over cost-effectiveness due to lack of appropriate evaluation data often leads to overestimating the clinical value of a new medicine, higher prices and concerns regarding who should fund the new medicine until more data becomes available (Fojo et al., 2014; Cohen, 2017; Pontes et al., 2020). Consequently, studies undertaken with data collected in routine care are becoming increasingly important as part of post-marketing activities to evaluate if the new medicines achieve the desired outcomes to support continued funding (Booth et al., 2019; Godman et al., 2021a).

In this context, real-world data collected outside randomized clinical trials (RCTs) is a powerful tool that can be used to generate robust real-world evidence to support future health authority decisions, including surrounding their funding and reimbursement (Cave et al., 2019; Martini et al., 2020). Real-world data collected in routine care can derive from a number of sources including hospital and pharmacy registers, electronic health records, administrative datasets, patient registers as well as population and healthcare surveys (OECD Health Division, 2019). Such data can complement RCTs to help assess the effectiveness of new medicines in routine clinical care versus their documented efficacy in trials (Khor et al., 2014; Kibbelaar et al., 2017; Kilburn et al., 2017; Zanotti et al., 2017; Eriksson et al., 2018; Baillie et al., 2020; Lemanska et al., 2020; Beachler et al., 2021; Lester et al., 2021; Nakayama et al., 2021). Real-world data has for instance been used in the evaluation of real-world outcomes of olaparib treatment for ovarian cancer in Sweden (Eriksson et al., 2018). Additionally, Frisk *et al.* (2018) in their follow-up studies using health authority databases in patients with chronic hepatitis C demonstrated an overall cure rate of 96% with second-generation direct-acting antivirals justifying continued funding (Frisk et al., 2018). Post-launch studies have also been undertaken confirming the effectiveness and safety of novel oral anticoagulants given initial concerns (Malmström et al., 2013; Mueller et al., 2019; Komen et al., 2021). We are also seeing generally an increase in the use of real-world data to support reimbursement and funding decisions (IQVIA, 2022).

*Cancer* registries have existed since the mid-20th century to monitor incidence, mortality and prevalence in populations and are increasingly being expanded and linked to other sources of data on medicine utilization as well as outcomes and effectiveness of oncology treatments (Siesling et al., 2015; Forsea, 2016; Booth et al., 2019). The availability of registries to monitor overall drug utilization in Europe has been investigated in both ambulatory care and hospitals (Ferrer et al., 2014; Sabaté et al., 2015). However, oncology medicines, especially new medicines, are a specific challenge since these are neither completely covered among prescription registries nor in the nationwide cancer registries (Siesling et al., 2015). Consequently, there is a need to document the availability of such resources among health authorities across Europe, as well as the type of data they collect, their robustness and applicability to inform continued funding decisions. This builds on ongoing European projects including the European Network of Centres for Pharmacoepidemiology and Pharmacovigilance (ENCePP) programe. ENCePP aims to strengthen research regarding the benefit-risk balance of medicines, including oncology medicines, in Europe by facilitating multi-centre, independent post-authorisation studies based principally on observational research. Alongside this, bringing together resources and expertise in pharmacovigilance and pharmacoepidemiology providing a platform for cross-collaborations (ENCePP, 2015). This also builds on any post-authorisation efficacy studies as part of registration with the European Medicines Agency (European Medicines Agency, 2022).

As a result, this study aims to outline the types of datasets that are available, especially among health authorities, regarding routinely collected patient-level data for oncology among different European countries. This includes what kind of patient-level data is routinely collected and the extent of its use from a health authority perspective. The objective being to better inform decision-making, including continued funding for new expensive oncology medicines. Additionally, to explore and understand the challenges and avenues for collaboration and data sharing across Europe principally among health authority personnel. This is important given the recognized complexities with the sharing of government and health authority data within and among countries. Complexities include issues surrounding security and privacy laws, technological challenges especially when combining different datasets (record linkage), organizational and financial concerns surrounding data entry, regulatory issues, limited government support and other political issues (Galetsi et al., 2019). However, we are aware there is a need to make patient-level data more available for research purposes across Europe to improve future patient care. We believe such discussions will contribute to improving accessibility, affordability and appropriateness of potential life-saving cancer therapies as more data becomes available.

## 2 Materials and Methods

### 2.1 Study Design

**FIGURE 1:**
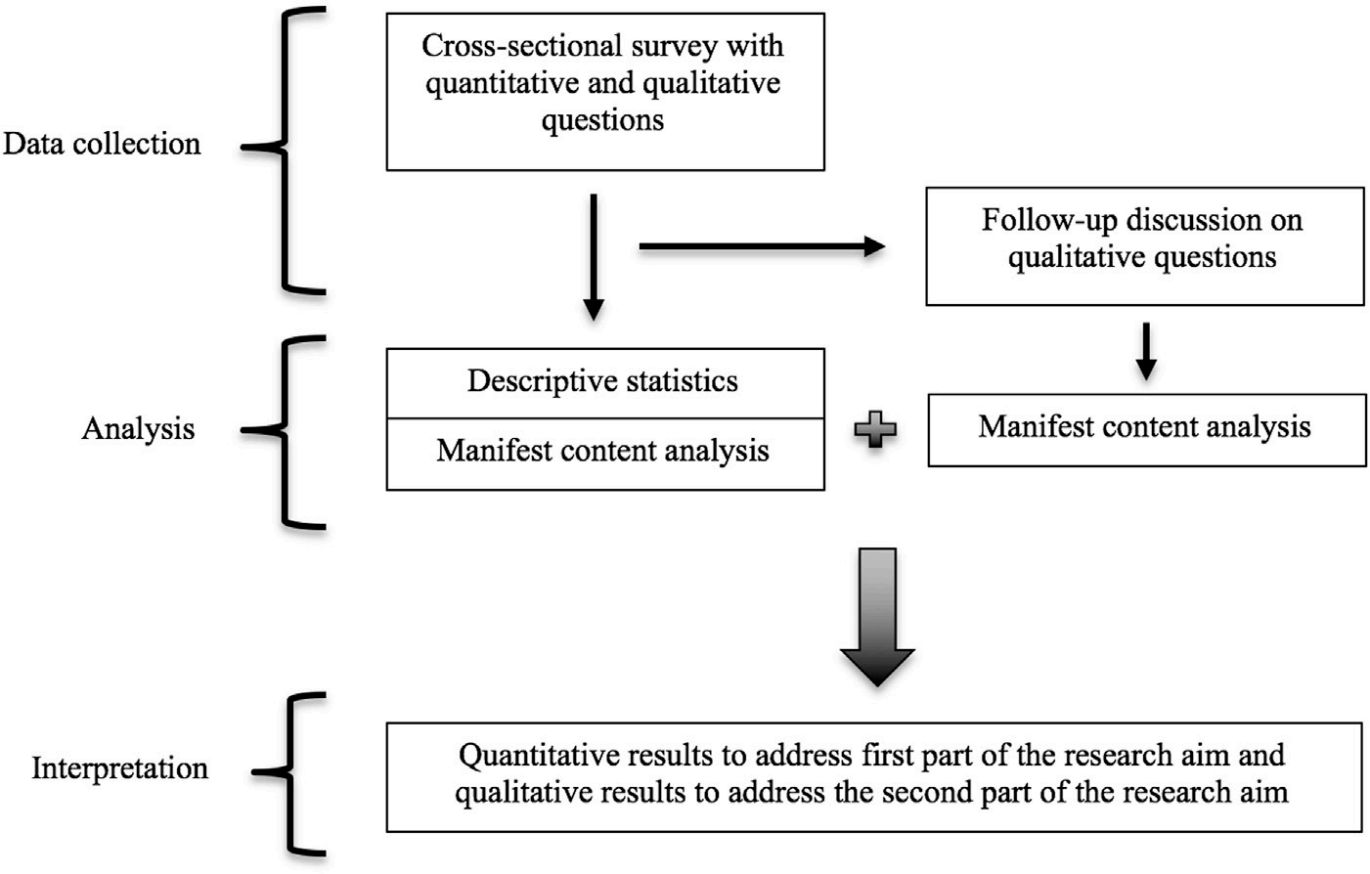
Visual representation of the study design steps for data collection, analysis and interpretation.

This study applied a mixed method approach consisting of a cross-sectional survey (Creswell and Clark, 2006), with the qualitative data collected simultaneously and integrated in the cross-sectional survey as open-ended questions (Figure 1). A follow-up discussion was undertaken after the cross-sectional survey data was collected to complement and further explore responses gathered form open-ended survey questions. Analogous mixed-method approaches have been used before by the authors and collaborators when conducting similar research on key topics across Europe (Wild et al., 2016; Vella Bonanno et al., 2017; Moorkens et al., 2017; Godman et al., 2019; Vella Bonanno et al., 2019; Gad et al., 2020; Godman et al., 2021a), as well as by others in various research fields (Taylor and Abernathy, 2014; Creswell and Hirose, 2019; Biermann et al., 2020).

### 2.2 Setting and Participant Sampling

**FIGURE 2:**
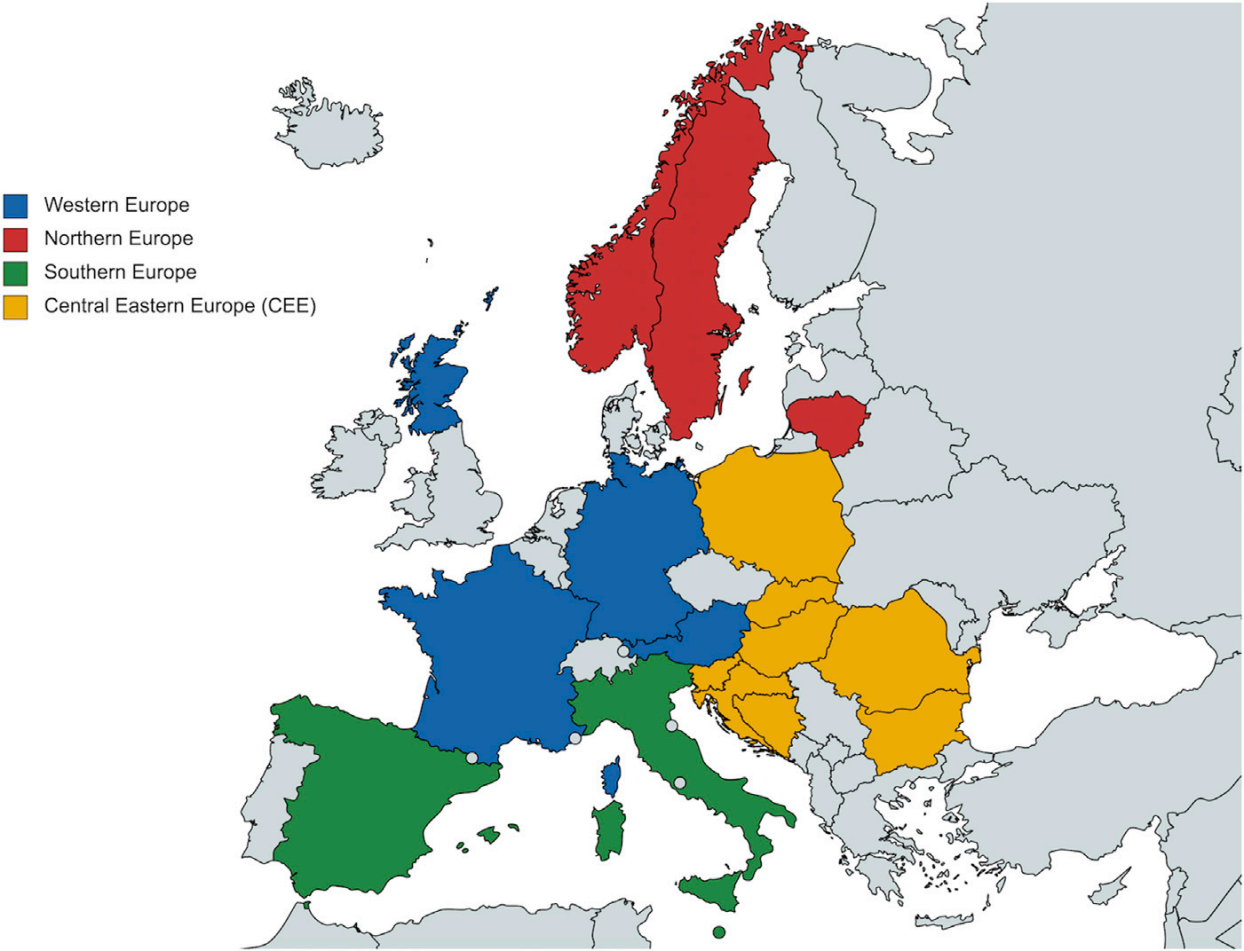
Map of countries included in the survey according to geographical region as defined by EU Vocabularies (European Commission, 2021). Map generated through MapChart (MapChart, 2021). It is important to note that Scotland and Catalonia are included in the study as independent entities from the respective countries (United Kingdom and Spain), with autonomous decision-making power including in the healthcare sector.

The survey was conducted among key stakeholders across the healthcare sector, especially health authority personnel and their advisers, from various European countries to represent different perspectives and experiences. Purposive sampling was considered the most appropriate strategy for this study as the main interest was to include key senior-level players that could provide the most up-to-date and relevant information and insights on the topic from the standpoint of their professional background. Consequently, key informants were purposefully selected to include clinicians, oncologists and particularly health authorities personnel and their advisers responsible for pricing, funding and reimbursement decisions for cancer medicines including new cancer medicines. They were also selected based on their country to include a wide range of geographical locations, population sizes, economic powers and health system organizations. Figure 2 and Table 1 illustrate the countries which were involved in the study, broken down by these different characteristics, which were considered important for the survey outcomes. In addition, snowball sampling was also used where appropriate to identify additional senior-level stakeholders suggested through the initial contacts.

**TABLE 1:** Country information broken down by population, economic power and type of health system.

Country	Population in 2020 (Millions) Eurostat, (2021a), Scottish Government (2021), Statistical Institute of Catalonia (2021a)	GDP per Capita in 2020 (€) Eurostat, (2021b), Statistical Institute of Catalonia, (2021b), Office for National Statistics (2021), The World Bank Data (2021)	Health System (European Health Observatory on Health Systems and Policies, 2021)
Austria	8.9	42,300	Social health insurance
Germany	83.2	40,490	Social health insurance
Scotland (United Kingdom)	5.5	33,744^a^	National health service
France	67.3	33,960	Social health insurance
Norway	5.4	59,180	National health service
Sweden	10.3	45,910	National health service
Lithuania	2.8	17,510	Social health insurance
Italy	59.6	27,780	National health service
Catalonia (Spain)	7.7	32,577^b^	National health service
Malta	0.5	25,310	National health service
Slovenia	2.1	22,310	Social health insurance
Slovakia	5.4	16,770	Social health insurance
Poland	39	13,640	Social health insurance
Hungary	9.8	13,940	Social health insurance
Croatia	4.1	12,170	Social health insurance
Romania	19.3	11,290	Social health insurance
Bulgaria	6.9	8,750	Social health insurance
Bosnia and Herzegovina	3.5^c^	5,031^d^	Social health insurance

a NB: GDP, for Scotland is from 2019 and was taken in GBP., It was converted to euros through the European Central Bank currency converter (European Central Bank, 2021) with the exchange rate for 2019.

b NB: GDP, for Catalonia is from 2019.

c NB: Population for Bosnia and Herzegovina is from 2019.

d NB: GDP, for Bosnia and Herzegovina was in US, dollars. It was converted to euros through the European Central Bank currency converter (European Central Bank, 2021) with the exchange rate for 2020.

Participants were identified through known research networks, such as the European branch of the International Society for Pharmacoepidemiology Special Interest Group for Drug Utilization Research (EuroDURG), as well as the Piperska group of policymakers and their advisers across Europe focusing on the rational use of medicines (Garattini et al., 2008; Sabaté et al., 2014). Many of these senior-level decision makers and academics, including some of the co-authors, have previously been involved through these networks in various cross-national studies on diverse areas of pharmaceutical policy, providing drug utilization and expenditure data, including on oncology medicines (Godman et al., 2014b; Moon et al., 2014; Godman et al., 2019; Pontes et al., 2020; Godman et al., 2021b). The stakeholders were invited by email to participate in the survey. The initial sample consisted of 56 participants selected through purposive sampling and an additional 4 were included through snowball sampling. In total 60 stakeholders across 28 countries were contacted and invited to take part in the study.

### 2.3 Data Collection

#### 2.3.1 Questionnaire

Data was collected through a structured questionnaire, with quantifiable questions including yes/no and a multiple choice format, as well as open-ended questions with a qualitative focus. A small pilot discussion was initially conducted with 6 key stakeholders, among the invited participants, from different European countries and regions (including Catalonia [Spain], Lithuania, Sweden, Poland and Scotland [the United Kingdom]) all of whom had a deep knowledge in the field. This resulted in an improved structuring of the survey as well as testing the feasibility and validity of the questions. A complete version of the questionnaire was developed following the pilot discussion, and was pretested with key selected informants to further refine the questions in terms of their clarity, focus and importance of the topics covered, to enhance the questionnaire validity and robustness.

The final survey was distributed in electronic format (through the Zoho Survey platform (Zoho Survey, 2021)) to the other identified stakeholders. The questionnaire was written in English and contained 20 questions, which were organized into four topics: 1) general availability of cancer medicines; 2) pricing and reimbursement systems; 3) types of databases collecting overall drug utilization and patient-level data in oncology; 4) future improvements and developments in data collection and data sharing (Supplementary File S1). The first two topics were included to gain understanding of the key issues surrounding the availability of cancer medicines and funding decisions, which will be followed-up in future research. The third and fourth topic more strictly pertain to this study and the outlined research aims. The responses were collected over a period of 2 weeks between March 29 and 14 April 2021.

#### 2.3.2 Focus Group Discussion

A focus group discussion was additionally conducted after the questionnaire data was collected to complement and consolidate understanding of the qualitative responses obtained to the open-ended survey questions. Participants for the discussion were selected among the survey respondents based on the extent of and need to clarify some of the open-ended responses provided. 19 respondents were invited via email, and six eventually took part in the focus group discussion, which was held through zoom. The discussion was moderated by two of the principal authors (BG and BW) due to their knowledge in this area to facilitate a stimulating and natural flow of the dialogue. The principal author (AP) was the assistant moderator and mainly responsible for taking notes and observations during the discussion. The session was videotaped after obtaining informed consent and the conversation was transcribed to use for analysis.

### 2.4 Data Analysis

#### 2.4.1 Quantitative

Using the questionnaire platform Zoho Survey and Microsoft Excel (version 16.16.27), quantifiable questionnaire data was analyzed with traditional descriptive statistics (frequencies, proportions, mean and median). When stakeholders from the same country provided contrasting answers, this was managed by checking back with the respondents for their interpretation of the questions and attempting to reach a consensus. However, this was not always possible. In these instances, contrasting responses within countries were maintained.

#### 2.4.2 Qualitative

Open-ended answers and the focus discussion transcript were analyzed with content analysis (Erlingsson and Brysiewicz, 2017), focusing on the manifest content. The content analysis focused on generating the meaning units, codes and categories that emerged from the open-ended questions and from the additional information obtained through the focus discussion.

### 2.5 Ethical Considerations

No ethical approval was sought for this project as the study did not involve handling of sensitive or confidential data and the issues discussed were not likely to bring any personal risk to the participants. In addition, the topic covered strictly pertained to the stakeholders’ professional competence and knowledge. Ethical considerations were made regarding completion of the questionnaire. This was addressed by providing comprehensive information to the stakeholders concerning the context and aim of the study. Participation was entirely voluntary, and participants indicated their consent to take part in the questionnaire form before providing their answers, with the option to decline to answer to any question or exit the questionnaire at any time. Furthermore, the voluntary option to include their name and contact details was included and participants were informed that this would be used only if they agreed to be further contacted for potential interviews. When conducting the focus group discussion, the participants’ informed consent was ascertained orally prior to recording the session. This is in accordance with national regulations and institutional guidelines and is in line with previous projects undertaken by the co-authors across a number of topics (Godman et al., 2014a; Vella Bonanno et al., 2017; Ferrario et al., 2017; Godman et al., 2018; Godman et al., 2019; Vella Bonanno et al., 2019; Gad et al., 2020; Godman et al., 2021a).

## 3 Results

### 3.1 Response Rate and Respondent Characteristics

Out of the initial sample of 60 stakeholders that were invited to take part in the questionnaire, a total of 25 stakeholders from 18 European countries (Figure 2) responded, resulting in a 42% response rate. The respondents represented a varied mix of different professional backgrounds across the healthcare settings (Table 2). In addition, a number of respondents were classified as “multiple affiliations” due to their involvements between health authorities, healthcare services, and academia. The results from the quantifiable survey responses are described in the following sections in terms of the proportion of participants who answered the questions as not all questions were answered by all 25 respondents.

**TABLE 2:** Respondent breakdown by professional setting.

Respondent Profession	Total n	Total %
Academic (research institute, university)	12	48
Healthcare professional (pharmacist, health services)	3	12
Health Authority (health insurance, social security, HTA^a^, medicine agency)	5	20
Multiple affiliations (university hospitals, academic institutions and health services or authorities)	5	20
**Total**	25	100

a HTA = Health Technology Assessment.

### 3.2 Overview of Oncology Datasets Across Countries

#### 3.2.1 Availability and Use of Databases

**FIGURE 3:**
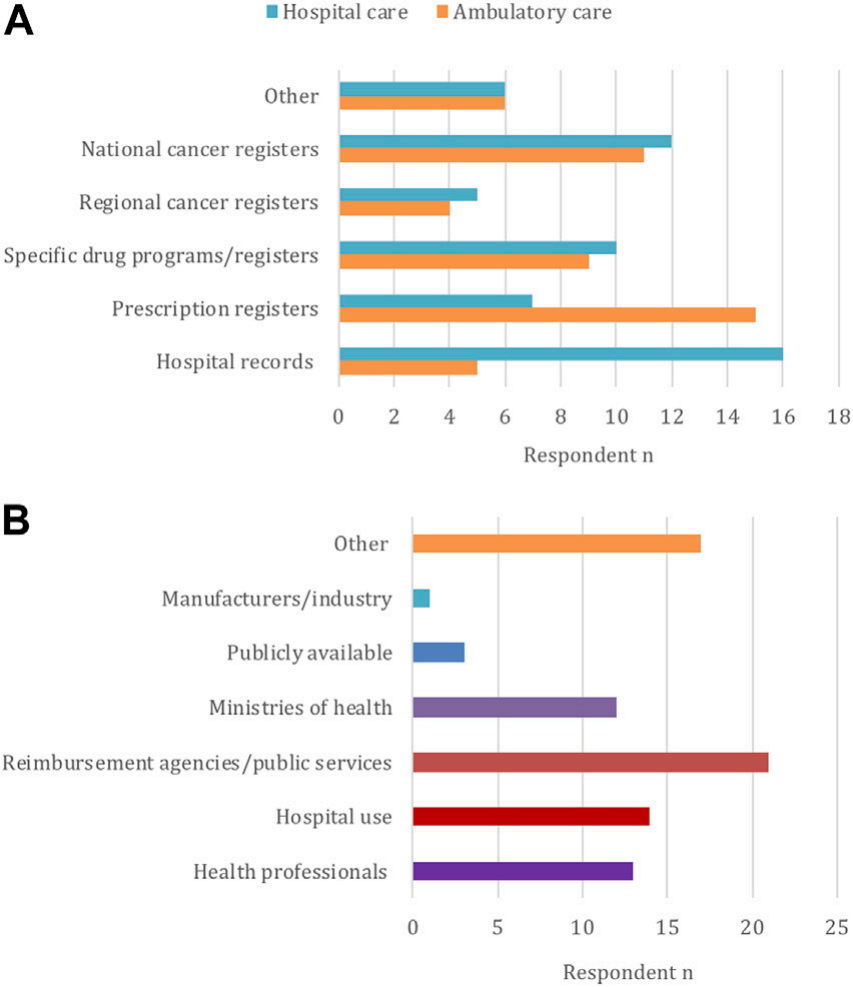
Types of databases for oncology (**(A)**, *n* = 24) and entities that may use the collected data (**(B)**, *n* = 25), according to the participants.

According to the responses from most stakeholders (*n* = 21/25), there are different types of organizations collecting drug utilization data across the countries, as displayed in Figure 3A. A summary of the situation concerning datasets in each country is also available in the Appendix (Supplementary File S2). Concerning hospital records, 76% (*n* = 16/21) of respondents said these are used to collect data for hospital medicines (inpatient care within their healthcare system), while this is less of a case for ambulatory care medicines (outpatient care) (24%, *n* = 5/21). In contrast, prescription registers were predominantly indicated for collecting ambulatory medicine data (71%, *n* = 15/21). Many respondents also documented the availability of national cancer registries that collect data for ambulatory (52%, *n* = 11/21) and inpatient care (57%, *n* = 12/21). This pertains to Bulgaria, France, Hungary, Malta, Norway, Poland, Scotland, Slovakia and Sweden (Supplementary File S2). A smaller proportion of respondents also indicated that regional cancer registries are employed to collect data in ambulatory (19%, *n* = 4/21) and hospital (24%, *n* = 5/21) care. Furthermore, some countries have specific drug programs or dedicated registers that collect data for oncology medicines both from hospital (48%, *n* = 10/21) and ambulatory care (43%, *n* = 9/21). This is the case for Hungary, Italy, Lithuania, Malta, Norway, Poland, Romania, Catalonia and Sweden (Supplementary File S2).

“Other” types of databases also exist as specified by 29% (*n* = 6/21) of respondents. Examples include the Scottish Prescribing Information Systems that records information for prescription medicines from community pharmacies as well as electronic prescribing for some hospital medicines; the National Health Insurance Fund and National Council on Pricing and Reimbursement in Bulgaria, which collect data and maintain registers for reimbursed and used medicines; and the French national claims data collected through the National Health Data System. In Sweden, register and clinical data can also be available through the Information Network for *Cancer* Care, a common platform to pool together different cancer registries (Supplementary File S2). Overall, 74% (*n* = 17/24) of stakeholders considered that databases that collect drug utilization data for oncology do not differ from structures that collect drug utilization data in general, with the exception of specific drug registries.

Concerning data access and use, 63% (*n* = 24/25) of respondents answered that there are specific regulations that limit data access and sharing, usually limited to data owners. According to the stakeholders’ responses (*n* = 25) (Figure 3B), databases or registries can be accessed or used by reimbursement agencies (84%, *n* = 21/25), hospitals (56%, *n* = 14/25), health professionals (52%, *n* = 13/25), and Ministries of Health (48%, *n* = 12/25). In contrast, data is less available for public access (12%, *n* = 3/25) and for pharmaceutical companies (4%, *n* = 1/25) (Figure 3B). 68% (*n* = 17/25) of participants also specified “other”, referring to possibilities of data availability for public use, research and academia, usually upon request and permission. This is the case for Germany, Austria, Sweden, France, Slovakia, Scotland, Catalonia and Hungary (Supplementary File S2).

#### 3.2.2 Characteristics of the Data Collected

**FIGURE 4:**
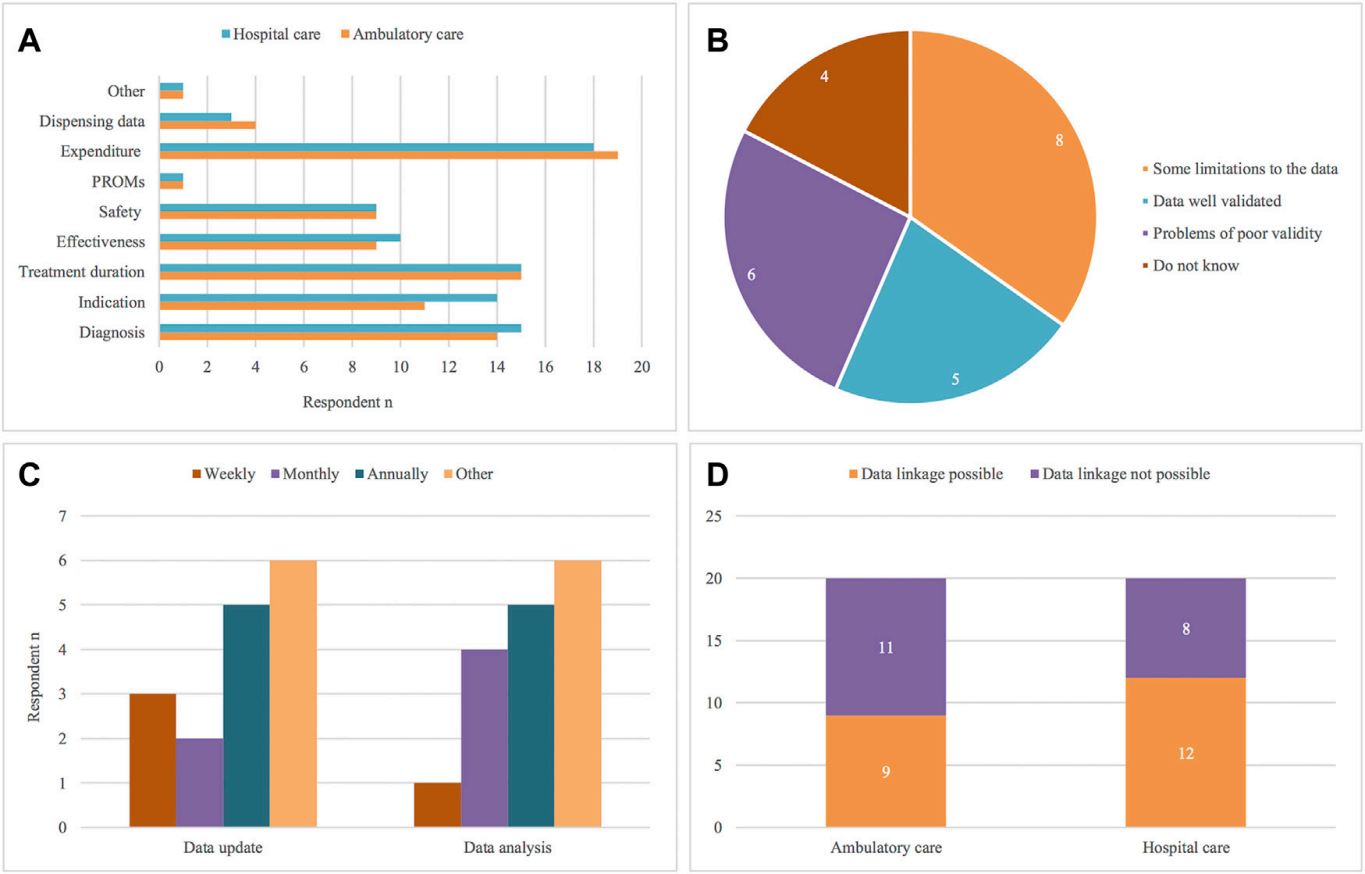
Types of oncology data recorded (**(A)**, *n* = 24), perceived data robustness and validity (**(B)**, *n* = 23), frequency of data update and analysis (**(C)**, *n* = 22) and possibilities for data linkage (**(D)**, *n* = 22), according to the participants. PROMs = Patient Reported Outcome Measures.

67% of respondents (*n* = 16/24) agreed that both individual-level and aggregated data is collected in their country. As shown in Figure 4A, the most widely available data in the majority of countries is medicine expenditure data, which is recorded both for medicines prescribed in ambulatory (90%, *n* = 19/21) and hospital care (86%, *n* = 18/21). A number of stakeholders also mentioned that data on diagnosis (ambulatory care: 67%, *n* = 14/21; hospital care: 71%, *n* = 15/21), indication (ambulatory care: 52%, *n* = 11/21; hospital care: 67%, *n* = 14/21) and treatment duration (71%, *n* = 15/21) is collected for both ambulatory and hospital settings.

As specified by 43% (*n* = 9/21) of the respondents, data on medicines safety such as adverse events is also recorded, as well as data on effectiveness measures such as survival, progression-free survival and quality of life (ambulatory care: 43%, *n* = 9/21; hospital care: 48%, *n* = 10/21). According to the responses, some countries only appear to collect safety data, such as Romania, Scotland and Sweden, or effectiveness data, as seen in Bulgaria and Lithuania. In contrast, both types of evidence were collected in Hungary, Italy, Norway, France, Poland, and Catalonia. Information on medicine dispensing is available in fewer countries for ambulatory (19%, *n* = 4/21) and hospital (14%, *n* = 3/21) care, as was stated by respondents from France, Hungary, Italy and Catalonia. Limited data on Patient Reported Outcome Measures is currently being collected among the involved countries, and this was indicated as available only by Scotland. The option “Other” was chosen when referring to instances where no precise schemes for data collection are established and the type of data recorded depends on individual registries or facilities collecting the data.

With regards to data robustness and validity, Figure 4B shows 35% (*n* = 8/23) of respondents answered that there are limitations with data robustness, and 26% (*n* = 6/23) that there are problems of poor validity. In contrast, 22% (*n* = 5/23) believed the data gathered is robust and well validated, whilst 17% (*n* = 4/23) had no knowledge or experience regarding this.

Another aspect of interest regarding the type of drug utilization data is how up to date the information collected is (Figure 4C). Concerning database update, 33% (*n* = 5/15) of respondents agreed this can occur annually, 20% (*n* = 3/15) weekly and 13% (*n* = 2/15) answered on a monthly basis. In terms of analyzing the data stored, 36% (*n* = 5.14) of respondents suggested the data is analyzed annually and 29% (*n* = 4/14) monthly, versus 7% (*n* = 1/14) saying this is undertaken on a weekly basis. Over 40% of respondents picked “other” as an option, referring to uncertainty of the answer, lack of knowledge or difficulty in providing a defined answer due to variation in how the data is collected and analyzed across databases.

Finally, the possibility of linking databases and registries across ambulatory and hospital settings within countries was also addressed in the questionnaire (Figure 4D). 45% (*n* = 9/20) of the stakeholders answered that linking datasets is possible in ambulatory care and 60% (*n* = 12/20) said so for databases in hospital settings. This pertains to Germany, Lithuania, Malta, Romania, Sweden, France, Catalonia, Hungary and Scotland. On the other hand, participants from Bosnia and Herzegovina, Slovenia, Croatia, Bulgaria, Italy and Slovakia answered linking datasets is not possible in their country neither in ambulatory care (55%; *n* = 11/20) nor hospital care (40%; *n* = 8/20).

### 3.3 Challenges and Opportunities for Collaboration and Improving Data Collection

The following key themes that were investigated through a qualitative analysis of open-ended questions and follow-up discussion are presented: 1) advantages and disadvantages of current data collection systems, 2) suggestions to improve data systems, 3) barriers and opportunities to cross-national collaboration.

#### 3.3.1 Advantages and Disadvantages of Current Data Collection Systems

**FIGURE 5:**
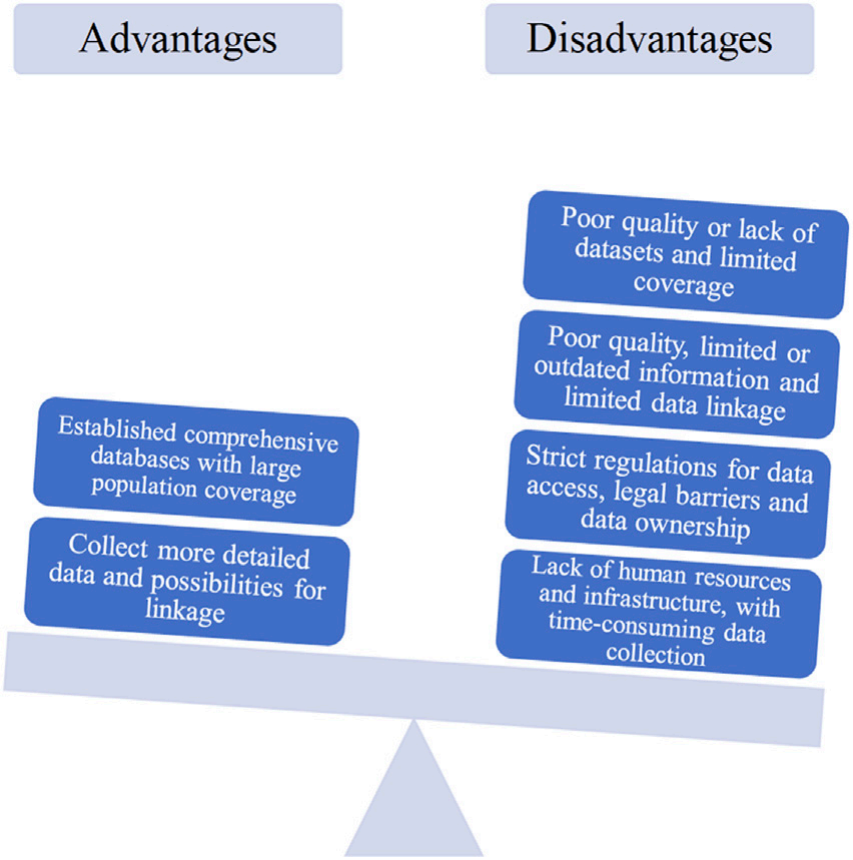
Main advantages and disadvantages of data collection systems for oncology identified by the participants.

**The established database systems**. The state of currently established databases was represented both as an advantage and disadvantage (Figure 5). In countries where comprehensive registries to collect drug utilization data across both ambulatory and hospital care settings are in place, this is seen as an advantage of current data collection systems, i.e. one that allows for the collection of ample information on medicine consumption, often with quite large population coverage. Nonetheless, in many countries there is a lack of registries and databases for patient-level and drug utilization data. In addition, even where available within one country, data collection systems are not always consistent in collecting data across regions, healthcare settings or therapeutic areas.

**Availability and extent of data collected**. A key drawback with the current data collection systems is that there is often limited data, mainly focusing on aggregated data for volumes and expenditure, compared to limited reporting of actual patient-level data on effectiveness, safety and patient outcomes measures (Figure 5). In line with this, the quality and detail of the evidence collected represents a concern as there are often gaps in the measures and variables that are recorded, which makes it difficult to accurately monitor and analyze treatment regimens, outcomes and adverse events (Figure 5). Most participants felt that a major hurdle to the efficient use and availability of data is that it is often not possible or very difficult to link data between datasets and healthcare settings within countries let alone across countries.

**Regulations for data access and use**. Closely linked to the availability and extent of data collected, many stakeholders suggested that strict regulations for data access and use represent further limitations in the data collection systems (Figure 5). The legal barriers in terms of data ownership and data protection exacerbate issues in accessibility of the data. Consequently, even when data is collected, it is often not available for analysis and use outside of the scope of hospitals, reimbursement agencies or other institutions responsible for gathering evidence and information for specific purposes.

**Resources for data collection**. A final issue that emerged as a drawback of current data systems is the resources - or lack thereof - needed for data collection (Figure 5). Many current information systems require oncologists, clinicians and physicians to enter the data manually, which represents a high additional workload and is time-consuming. In addition, the lack of dedicated staff, financial resources and IT infrastructure to speed and facilitate data recording can result in data not being accurately recorded and in low reporting rates, further exacerbating issues with data quality, validity and robustness.

#### 3.3.2 Suggestions to Improve Data Systems

**Improving policies and guidelines for data collection**. Participants suggested the establishment of better guidelines and regulations for data access as a step towards improving data systems. Namely, there is pressing need for more transparency in publishing data and strengthening opportunities to use the available evidence for analysis and observational studies. However, mindful of existing security and privacy regulations within countries in terms of data collection and analysis. The promotion of further incentives for healthcare professionals to collect and provide detailed routine clinical data to health authority and other key stakeholder groups is also a potential step to improve the current datasets. Building on comparisons and successful examples from different countries through health authority cooperation is also a key step for future improvements in the prompting of real-world evidence, as well as developing common data models to pool and analyze data from different sources both within and among countries.

**Investing in databases**. Stakeholders discussed how addressing guidelines for data collection and access should be accompanied by further investment in current and future databases, with many countries requiring the introduction of registries and information systems where they are not available at present. Moreover, previously established databases necessitate expanded coverage at national and regional levels, and encompassing different healthcare settings including hospital and ambulatory care, as well as promote systems and common data models that allow information to be more easily linked across databases and healthcare settings.

**Allocating resources for data collection**. A further area that was highlighted by the participants was the importance of allocating more resources to data collection in terms of having dedicated people and competent staff other than medical professionals involved in reporting data to alleviate workloads. In addition, enhanced resources and infrastructure for automatization in data capture and entering would also simplify and improve the data collection process.

#### 3.3.3 Barriers and Opportunities for Cross-National Collaboration

**FIGURE 6:**
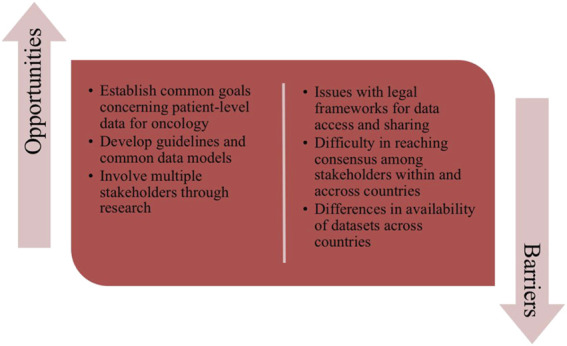
Key opportunities and barriers outlined by the participants for cross-country collaborations to improve data collection systems for oncology across Europe.

**Challenges of promoting collaborations in the short term**. Whilst there is agreement that cross-country collaboration is an important factor to promote the collection of meaningful data especially in the cancer field, the general opinion reflects current barriers and challenges that often hinder efficient cooperation and improvements (Figure 6). These include the many differences in the availability and structures of health authority and other databases across countries as well as how health systems are organized in the provision of care. Consensus is that much has to be achieved first within individual countries to improve their data collection before potentially strengthening collaborations cross-nationally. On this front, the engagement of multiple stakeholders from different professional and healthcare settings is considered a key opportunity to share knowledge and to obtain meaningful patient-level data for oncology. Nevertheless, this can also represent a barrier to collaboration as it can be difficult to reach consensus especially with important organizational issues as well as potential involvement with commercial organizations*.* Moreover, issues with legal frameworks to data access and sharing can also hinder the establishment of cross-national cooperation for common datasets to improve availability of individual-level data across Europe.

**Recommendations and legislations**. To facilitate engagement at the European level, stakeholders consider the most feasible way forward involves maintaining and promoting further engagement in cross country research projects and networks. This could foster a better understanding of the situation concerning the availability of patient level-datasets for oncology across Europe, and identify common visions and targets to encourage smoother cooperation between health authorities and others across countries through the establishment of guidelines and common models for data collection, analysis and data-sharing.

### 3.4 *Cancer* Medicine Availability, Pricing and Reimbursement

Various key cancer medicines were mentioned to be the current focus across countries in terms of their prices, expenditure and patient use. As this was an open question and not answered by all respondents, it is difficult to quantify the medicines. However, an overview of the different oncology medicines mentioned is available in Supplementary File S3 and will be the subject of future research projects.

Overall, a wide variety of medicines was specified for individual countries. The following medicines were mentioned by multiple countries: Ibrutinib, Nivolumab, Paclitaxel, Palbociclib, Pembrolizumab, Trastuzumab which suggests these oncology medicines could be of common interest in terms of priority therapeutic indications, consumption and budget concerns. We will be following this up in future research projects.

Among most countries, funding of oncology medicines is regulated at the national level both for ambulatory (88%, *n* = 22/25) and hospital (64%, *n* = 16/25) medicines. In fewer instances, funding is managed at the regional level (for hospital medicines) or at both levels (Supplementary File S4A). MEAs or other risk-sharing arrangements are commonly used mechanisms to establish pricing agreements, with 82% (*n* = 18/22) respondents indicating there are 5 or more operating nationally, and 56% (n = 5/9) regionally (Supplementary File S4B). MEAs and other similar schemes involve confidential discounts (67%, *n* = 16/24), price: volume agreements (63%, *n* = 15/24) and price: cap agreements (58%, *n* = 14/24), and to a lesser extent outcome schemes (46%, *n* = 11/24). 64% of respondents also specified “other” arrangements, including pay-back schemes, budget caps, procurement by tendering, conditional reimbursement among others (Supplementary File S4C).

## 4 Discussion

Our findings show that there is appreciable variation and fragmentation in the availability of registries and databases, including health authority/health insurance company databases, to collect patient-level data in oncology across Europe. This includes cancer registries, prescription registers and hospital records, as well as registries for specific drug programs, which is typically collected data for use in the context of health authorities such as reimbursement agencies, Ministries of Health, as well as hospitals. There are also differences in the type of data collected, where aggregate expenditure data is the most widely available. However, patient-level data concerning diagnosis, treatment and indication, as well as effectiveness and safety of medicines, is collected to a lesser extent, particularly concerning outcome measures.

Our study also highlights the main concerns associated with current patient-level datasets for oncology. These include the lack of comprehensive registries across countries and healthcare settings, and the limited evidence available on effectiveness, safety and patient outcomes of new cancer medicines, especially with regards to medicines prescribed for inpatients in hospitals. Major hurdles with data ownership limit data accessibility and use, as well as possibilities for linking datasets, and the data collection process is time-consuming for health professionals who need to compile registries. This requires more financial resources to invest in dedicated staff and better information systems to facilitate the recording of data. Fostering cross-national collaboration among health authorities and establishing better guidelines for transparency, publishing and strengthening data sharing are an important aspect moving forward.

The variation and fragmentation in the availability of databases and type of data collected is in part influenced by the different types of healthcare financing systems such as national health services or insurance-based models (Table 1), how different countries manage funding and reimbursement at the national or regional level, and how this can vary for medicines dispensed in ambulatory or inpatient care (Wilking and Jönsson, 2005). These differences are also reflected in the varying patterns in uptake and availability of new oncology medicines that have been observed across Europe (Wilking and Jönsson, 2005; Mayor, 2016; Hofmarcher et al., 2019). Furthermore, different funding mechanisms are increasingly being adopted across Europe, including MEAs and risk-sharing schemes, to address the affordability issue of new cancer medicines, which will likely influence their uptake and the type of data collected to support these schemes (Pauwels et al., 2017; Godman et al., 2021a). Consequently, funding policies and health financing structures may impact the different types of data reporting systems available. The many sources of patient-level data observed across Europe, as well as the scope and quality of data gathered, may also reflect the incentives there are for its collection and how the data is subsequently used. For instance, in countries where health data is owned by health insurances and reimbursement agencies, the type of data available might focus on expenditure and consumption and be limited for the region covered by that service; consequently, it is more difficult to collect data on a national scale (OECD Health Division, 2019). In contrast, some countries with nationally or regionally organized health systems are more advanced in terms of registries and electronic health records with large population coverage, allowing for information to be linked and integrated across care settings (OECD Health Division, 2019).

Our findings concerning the challenges and opportunities to improve data collection accentuate the many concerns associated with the current availability of oncology datasets among health authorities and others, and the type and quality of clinical data being collected. They also underline how, despite the availability of technology and information systems, practice and reality are quite different from expectations that establishing comprehensive cross-country patient-level datasets are easily feasible. As highlighted, fragmentation of registries and databases is an issue across and within countries, and reflects the different capacities, financial and technological resources available to establish detailed and accurate data networks (Montouchet et al., 2018). Electronic health records and registries might be specific to certain healthcare settings but not available in others, and there are little guidelines, criteria and lack of common data models to ensure uniform collection of data within countries, let alone across borders. Furthermore, there are still significant hurdles restricting access and secondary use of patient data for research and healthcare purposes (Galetsi et al., 2019), even for researchers working with health authority data to address key health policy issues. These include barriers due to ownership and lack of transparency in data use, as well as data privacy and protection laws, hindering the possibility to extensively link datasets to obtain and harness routine data to inform policy decisions (Montouchet et al., 2018; OECD Health Division, 2019).

Nevertheless, there are examples of positive changes moving forward, reflected by a number of initiatives across Europe. The Scottish Cancer Medicines Outcome Program (CMOP) is a noteworthy example in pooling together different datasets available to make better use of data for safety, effectiveness and treatment outcomes for the different oncology medicines (CMOP, 2020). The program has demonstrated success in linking registries and electronic records, as well as collecting more patient-level data on quality of life and Patient Reported Outcome Measures (Baillie et al., 2020; CMOP, 2020). In addition to CMOP in Scotland, another interesting initiative is the Systemic Anti-Cancer Therapy datasets in the United Kingdom, which routinely collects and reports data on cancer patients, regimens and treatments outcomes through the National Health Service (Bright et al., 2020). Its wide population coverage and ability to link across different routine care databases within the National Health Service are key strengths that allow for collection of comprehensive evidence to support decision-making on delivery of care and complement RCT evidence for medicines with uncertainty over their clinical value, to better inform funding decisions (Bright et al., 2020). Along the same lines, the Catalan Health Services experience with registries allowed for the consolidation of a Patient and Treatment Registry across all public hospitals in Catalonia, collecting exhaustive information on treatments, indications and clinical variables and can be linked to other registries (Roig Izquierdo et al., 2020). The information collected is analysed and integrated in decision-making concerning MEAs, re-assessment of medicines and indicators based on effectiveness to assess quality and rational use of medicines. This also allows health authorities to discuss the results with hospitals and clinicians with respect to their practices and to review and follow-up on the Catalan Health Services recommendations (Reyes-Travé et al., 2021). Real-world data initiatives have also taken shape in the Scandinavian countries. For instance, in Sweden studies concerning ovarian and prostate cancers have demonstrated the value of harnessing real-world data from registries and health records to investigate and understand the longer-term outcomes of cancer treatments (Eriksson et al., 2018; Beckmann et al., 2021). Nonetheless, it is interesting to note that despite the long history of Nordic countries with establishing cancer registries (Pukkala et al., 2018), there seems to be no clear lead in real-world data initiatives compared to other countries mentioned. In contrast, promising activities are arising across European regions, creating opportunities for comparisons and a shared learning environment.

### 4.1 Strengths and Limitations

The involvement of key senior-level players representing various professional backgrounds in different healthcare settings across European countries is a major strength of the study alongside the wide range of countries included in this study. Nevertheless, this study has several limitations. Since the intention was to select specific stakeholders in individual countries no sample size calculation was conducted as this was not considered appropriate. Nonetheless, this, along with the relatively small sample of 25 stakeholders, limits the generalizability of the quantitative findings. Additionally, as the survey contained different questions spanning medical practice, funding and policy, respondents’ background may have limited the extent of responses for some questions over the others. Furthermore, it is important to consider that the responses provided are based on the stakeholders’ knowledge and experience in the field, which may have biased the interpretation of survey questions. For instance, participants from a health authority perspective are usually more informed regarding issues of policy and funding, and may have more knowledge regarding datasets collecting information on expenditure, consumption and volume rather than looking at patient outcomes. On the other hand, oncologists, clinicians, pharmacists and other healthcare professionals might be more knowledgeable with issues concerning the effectiveness and safety of different oncology medicines and the situation concerning data collected at the patient-level.

Concerning the qualitative aspect of the methodology, this principally allowed an opportunity to gain a general overview and understanding regarding the main issues and opportunities to improve datasets in the future. In view of this, the open-ended questions and discussion was potentially limited in terms of depth of understanding and reaching saturation, and perhaps further group discussions or interviews with additional stakeholders could have yielded additional knowledge. Consequently, the objective and scope did not allow for an extensive exploration of this topic nor an in-depth review of all databases available in each country. Despite these limitations, the findings are believed to be valid given the seniority and range of different stakeholders approached across Europe.

### 4.2 Conclusion and Future Implications

We believe the data presented here are the most recent and updated knowledge at present as provided among European countries involving key stakeholder groups, but this could quickly change in the near future. Nevertheless, this study has important implications for the future of real-world data collection for oncology, particularly as this area will likely develop as a high priority for policy agendas. With the increasing number of high-priced medicines that are launched with immature data, expenditure and opportunity costs need to be accounted for by payers to balance finite healthcare budgets with the necessity to provide access to safe and cost-effective cancer medicines. These concerns can be addressed by collecting more data on the performance of a new medicine in routine care, to re-define funding decisions and better allocate resources for healthcare (Barrett et al., 2006; Godman et al., 2021a; Malmström et al., 2013). Consequently, through this study we highlight the imperative need to move forward in collecting standardized datasets for oncology.

To achieve this, a key step will be to continue involving multiple health authority and other stakeholders across the healthcare sectors and build a more common understanding of the value of real-world data on a European level in order to establish the necessary technology, infrastructure and resources to incentivize data collection for oncology and improve its quality and availability across countries. In line with this, building on current initiatives and promoting European-wide cooperation and research engagements will lay the ground for defining clear and common guidelines for implementing data use and develop information platforms for data sharing and linkage (Montouchet et al., 2018; OECD Health Division, 2019). Overall, this study has important relevance in terms of pharmaceutical policy, as the collection of more robust and comprehensive data on patient outcomes, drug performance, effectiveness and safety can help re-shape pricing, reimbursement and funding policies, regulatory processes, drug utilization policies as well as promote accessibility, affordability and appropriateness of new cancer medicines.

## Data Availability

The raw data supporting the conclusions of this article will be made available by the authors, without undue reservation.
