# Supplementary material for: Challenges and Opportunities With Routinely Collected Data on the Utilization of Cancer Medicines. Perspectives From Health Authority Personnel Across 18 European Countries
Source: Front Pharmacol. 2022 Jun 16;13:873556. doi: 10.3389/fphar.2022.873556 (PMC9295616; doi:10.3389/fphar.2022.873556)
Supplement: Supplementary file 1 [file Table1.docx]

Supplementary File 2

**Supplementary table 1.** Summary information of pricing, reimbursement and availability of datasets for each country.

| **Country** | **Pricing and reimbursement** | **Overview of patient-level datasets** |
| --- | --- | --- |
| Austria | **Separate procedure for ambulatory and hospital medicines:** Yes.  **Regulatory level:** national for ambulatory medicines, regional for hospital medicines.  **Key responsible authorities:** for ambulatory care medicines the DVSV (social health-, accident- and pension insurance institutions); for  hospital medicines can be on a federal state level (Hospital Corporations), but also on a hospital level.  **MEAs/risk-sharing arrangements:** nationally operating mechanisms include confidential discounts and price: volume agreements.  **Challenges and opportunities with MEAs:** The main challenge of these arrangements is the administrative burden and data confidentiality. | **Facilities collecting data for cancer care:** For the ambulatory sector billing data is available and collected routinely. In some federal states specific registries can be in place.  **Available for use and analysis by:** Health professionals, hospital use, reimbursement agencies/public services, Ministries of Health, and academia upon request.  **Type of data:** aggregated (this mainly in the outpatient sector) and individual level data collected. The most available and routinely collected in ambulatory care is data on expenditure. Diagnosis and indication is also available for hospital care.  **Specific regulations for data access:** Data is owned by payers, subsets can be made available upon specific request and in relation to specific research question.  **Data linkage between registers:** no linkage as of yet.  **Data robustness and validity:** there are issues with data quality and the level of detail in the data collected is poor. Concerning the outpatient sector, the Austrian Social Insurance has access to comprehensive, robust and valid billing data for ambulatory medicines, including oncology ones. However no patient-level data.  **Database update and analysis:** for routine billing data for ambulatory medicines databases are updated and analyzed monthly.  **Databases established since:** N/A |
| Bosnia and Herzegovina (Republic of Srpska) | **Separate procedure for ambulatory and hospital medicines:** No  **Management level:** national for ambulatory and hospital medicines.  **Key responsible authorities:** the Health Insurance Fund of the Republic of Srpska, responsible for reimbursment and pricing  The Agency for Medicinal Products and Medical Devices Bosnia and Herzegovina (ALMBIH), responsible for defining the maximum wholesale price of medicines.  **MEAs/risk-sharing arrangements:** price: volume agreements.  **Challenges and opportunities with MEAs:** they enable the system to provide new medicines for the patients taking into account current financial capabilities. However, the number of patients higher than current financial capabilities, still rather high prices after negotiations. | **Facilities collecting data for cancer care:** hospital medical records, Public Health Institute of the Republic Srpska (collects all hospital and ambulatory medicines utilization data and keeps a national cancer register), Health Insurance Fund of the Republic of Srpska (collects reimbursed medicines utilization data).  **Available for use and analysis by:** health professionals, hospital use, Health Insurance Fund of the Republic of Srpska, Ministry of Health and Social Welfare of the Republic of Srpska , academia. Public could apply to access some of the data..  **Type of data:** aggregated and individual level, mainly on expenditure.  **Specific regulations for data access:** no.  **Data linkage between registers:** no.  **Data robustness and validity:** N/A  **Database update and analysis:** N/A  **Databases established since:** N/A |
| Bulgaria | **Separate procedure for ambulatory and hospital medicines:** no.  **Management level:** nationally for both ambulatory and hospital medicines.  **Key responsible authorities:** National Council of pricing and reimbursement of medicinal products (NCPRMP**).**  **MEAs/risk-sharing arrangements:** nationally operating mechanisms involve outcome schemes, confidential discounts, price cap agreements, budget cap, payback, therapeutic effect monitoring.  **Challenges and opportunities with MEAs:** allow for cost-containment but difficult to monitor performance of new medicines. | **Facilities collecting data for cancer care:** hospital records and national cancer registers collect data for inpatient medicines; the national health insurance fund and national council on pricing and reimbursement also collect data for ambulatory medicines.  **Available for use and analysis by:** health professionals, hospital use, reimbursement agencies. Some data could be publicly available.  **Type of data:** both aggregated and individual-level data on diagnosis, indication, treatment duration, effectiveness and expenditure.  **Specific regulations for data access:** no.  **Data linkage between registers:** no.  **Data robustness and validity:** data collected quite limited, mainly collected at hospital level.  **Database update and analysis:** monthly/annual update and analysis.  **Databases established since:** 2003 for prices and reimbursement and since 1952 national cancer register. |
| Croatia | **Separate procedure for ambulatory and hospital medicines:** no.  **Management level:** national for both ambulatory and hospital medicines.  **Key responsible authorities:** The Croatian Agency for Medicinal Products and Medical Devices and the Croatian Health Insurance Fund.  **MEAs/risk-sharing arrangements:** nationally operating mechanisms include confidential discounts, price: volume agreements, price cap agreements.  **Challenges and opportunities with MEAs:** major challenge of monitoring outcomes. | **Facilities collecting data for cancer care:** Collected mainly in hospital records for hospital medicine.  **Available for use and analysis by:** Health professionals, reimbursement agencies/public services.  **Type of data:** aggregated data. Includes data on diagnosis, indication, treatment duration and expenditure.  **Specific regulations for data access:** no.  **Data linkage between registers:** There are no registries or databases that can be linked and data is not very accessible.  **Data robustness and validity:** There are validity problems with the data collected.  **Database update and analysis:** N/A  **Databases established since:** N/A |
| France | **Separate procedure for ambulatory and hospital medicines:** yes.  **Management level:** national for both ambulatory and hospital medicines.  **Key responsible authorities:** HAS (High Health Authority)  **MEAs/risk-sharing arrangements:** in operation nationally. Include mainly price: volume agreements.  **Challenges and opportunities with MEAs:** little data on appropriate use (appropriate indications, appropriate prescribing, precautions for use, appropriate use by patients). | **Facilities collecting data for cancer care:** for both ambulatory and hospital medicines data is recorded through hospital records, prescription registers, national cancer registers and National claims data – the SNDS (National Health Data System), managed by the CNAM (French NHS). There is more efforts to collect data in oncology on detailed use of medicines, compared to other areas.  **Available for use and analysis by:** Hospitals, reimbursement agencies/public services, Ministries of Health.  **Type of data:** aggregated and individual-level data on treatment duration, safety, effectiveness, expenditure and dispensing data. Algorithms are used to identify specific diagnoses and indications.  **Specific regulations for data access:** The national claims data are officially publicly available. In practice, there is long and complex procedures to access the data.  **Data linkage between registers:** linking registers between ambulatory and hospital care is feasible but long and complex process.  **Data robustness and validity:** some limitations in the data collected, notably quality of diagnosis uncertain.  **Database update and analysis:** generally database are updated annually but analysis carried out on weekly basis.  **Databases established since:** N/A |
| Germany | **Separate procedure for ambulatory and hospital medicines:** Yes  **Management level:** National for ambulatory medicines, regional for hospital medicines.  **Key responsible authorities: the** Joint Federal Committee (G-BA) is responsible for reimbursement and pricing is negotiated by health care funds (GKV-Spitzenverband). Hospitals negotiate their own prices.  **MEAs/risk-sharing arrangements:** in operation at regional and national level. These include outcome schemes, confidential discounts and price-cap agreements.  **Challenges and opportunities with MEAs:** can lower expenditure for very high-priced drugs, but issues with lack of transparency; pitting health care funds against each other; introduction of drugs with doubtful added therapeutic benefit. | **Facilities collecting data for cancer care:** data is collected for ambulatory cancer medicines through prescription registers, national and regional cancer registries. Hospital medicine data is collected through hospital records and through regional cancer registers.  **Available for use and analysis by:** hospitals, health professionals, reimbursement agencies and public services, Ministries of Health.  **Type of data:** Individual-level data. In ambulatory care, diagnosis and expenditure data is the most available; in hospital care this depends on the hospital record.  **Specific regulations for data access:** Data can also be accessed for research following application to the Federal Institute of Drug and Medical Devices (DIMDI).  **Data linkage between registers:** Data can be linked across registries to some extent, for research purposes.  **Data robustness and validity:** Data robustness varies, with some limitations  **Database update and analysis:** timescales for update and analysis vary across databases.  **Databases established since:** 1926. |
| Hungary | **Separate procedure for ambulatory and hospital medicines:** In Hungary oncology medicines are all reimbursed, the patient has to pay a “package  fee” for outpatient medicines, but not for inpatients medicines. Outpatient oncology medicines are financed via public procurement and named patient base program, inpatient therapies are financed via Diagnosis-Related Group system.  **Management level:** national for both ambulatory and hospital medicines.  **Key responsible authorities:** National Health Insurance Fund of Hungary (NEAK),  Ministry of Health, HTA Body  Medical Professional College.  **MEAs/risk-sharing arrangements:** nationally operating mechanisms involve outcome schemes, confidential discounts, price: volume agreements, price cap agreements, box fee.  **Challenges and opportunities with MEAs:** They increase patient access to beneficial therapies. Main challenges of budget sustainability, uncertainty minimalization - real world evidence, administrative burden. MEAs are mandatory for every new innovation (also for new cancer therapies). | **Facilities collecting data for cancer care:** ambulatory and hospital medicine data is collected through prescription registers, specific drug programs, national cancer registers. Additionally hospital records for inpatient medicines.  **Available for use and analysis by:** reimbursement agencies, ministries of health, health professionals and HTA bodies with special permission.  **Type of data:** aggregated data is easier to collect and access than individual-level data on diagnosis, indication, treatment duration, effectiveness, safety, expenditure, dispensing for both ambulatory and hospital medicines.  **Specific regulations for data access:** strict legal background for access and sharing. The National Fund has open-access database regarding all ambulatory drug consumption, but more difficult to reach the hospital drug use. If somebody is interested has to find partners for cooperation, but mostly on hospital not individual level.  **Data linkage between registers:** expert oncology groups work together to facilitate linkage of databases in ambulatory and hospital care.  **Data robustness and validity:** there are some issues with robustness and data validation.  **Database update and analysis:** data collection is continuous in terms of consumption (packages) of different drugs, including oncology drugs, but there is no obligation for detailed analysis. Expenditure is analyzed and provided monthly. Inpatient care databases updated weekly and analyzed monthly, outpatient care databases updated daily/weekly/monthly (depending on the hospital and therapeutic field) and usually analyzed monthly.  **Databases established since:** 1999 |
| Italy | **Separate procedure for ambulatory and hospital medicines:** no.  **Management level:** national for both ambulatory and hospital medicines.  **Key responsible authorities:** The Italian Medicines Agency (AIFA).  **MEAs/risk-sharing arrangements:** nationally operating schemes include outcome schemes, confidential discounts, price: volume agreements, price cap agreements. MEAs include performance-based risk sharing schemes (Payment by result, Risk sharing, Success fee) and financial based schemes (Cost sharing, Capping). | **Facilities collecting data for cancer care:** prescription registers, specific drug programs/registers collect data for ambulatory and hospital medicines. There are also monitoring registers that allow access to treatments that need to be monitored (such as newly approved medicines) for their use and their safety, effectiveness and outcomes.  **Available for use and analysis by:** reimbursement agencies, Ministry of Health.  **Type of data:** both aggregated and individual-level data is collected. Types of data include diagnosis, indication, treatment duration, effectiveness, safety, expenditure, dispensing.  **Specific regulations for data access:** no, there is limited accessibility.  **Data linkage between registers:** no, but potentially local initiatives of data linkage at the hospital level**.**  **Data robustness and validity:** N/A  **Database update and analysis:** N/A  **Databases established since:** N/A |
| Lithuania | **Separate procedure for ambulatory and hospital medicines:** Yes.  **Management level:** national for both ambulatory and hospital medicines.  **Key responsible authorities:** National Health Insurance Fund, the Ministry of Health and National Medicines Agency.  **MEAs/risk-sharing arrangements:** in operation nationally. Include outcome schemes, confidential discounts, price: volume agreements, price cap agreements.  **Challenges and opportunities with MEAs:** challenges of growing expenditure, patient health data management for outcome-based schemes and the unclear situation with information about medicines prices. | **Facilities collecting data for cancer care:** Both ambulatory and hospital medicine data is collected through hospital records, prescription registers and national and regional cancer registers. Hospital medicines are also included in specific drug programs/registers.  **Available for use and analysis by:** data owners – hospitals, national or regional reimbursement agencies/public services such as NHIF, National e-health database.  **Type of data:** aggregated and individual-level. Databases record data on diagnosis, indication, treatment duration, expenditure, and effectiveness is also recorded for hospital care medicines.  **Specific regulations for data access:** There are no regulations for data sharing and secondary health data record usage.  **Data linkage between registers:** Data can be linked between registries/records in ambulatory and hospital setting.  **Data robustness and validity:** data robustness is limited  **Database update and analysis:** Databases are updated weekly but there are no guidelines for analysis and this is carried out annually.  **Databases established since:** 2002 |
| Malta | **Separate procedure for ambulatory and hospital medicines:** no.  **Management level:** national for both ambulatory and hospital medicines.  **Key responsible authorities:** Medicines listed on the Public Formulary List and supplied through the public health services are procured centrally by the Centralised Procurement Supplied Unit. These are given free to the patients who fulfil the entitlement and clinical criteria.  **MEAs/risk-sharing arrangements:** In operation nationally. Include outcome schemes, price: volume agreements, price cap agreements and procurement by tendering.  **Challenges and opportunities with MEAs:** Once a patient is started on treatment it will not be possible to stop treatment if the patient insists on continuing. For some medicines it is difficult to do measurement of effectiveness in an objective manner. | **Facilities collecting data for cancer care:** ambulatory and hospital medicine data is collected through hospital records, prescription registers, national cancer registries and specific drug programs. There are also lists of patients for specific medicines for which approval is required.  **Available for use and analysis by:** hospitals, reimbursement agencies/public services, Ministries of Health - only on request for specific purposes.  **Type of data:** individual-level and aggregated data is collected on diagnosis, indication and expenditure for both ambulatory and inpatient medicines.  **Specific regulations for data access:** Freedom of Information Legislation applies.  **Data linkage between registers:** There is possibility for data linkage in ambulatory and hospital settings.  **Data robustness and validity:** N/A  **Database update and analysis:** N/A  **Databases established since:** N/A |
| Norway | **Separate procedure for ambulatory and hospital medicines:**  **Management level:** national for both ambulatory and hospital medicines.  **Key responsible authorities:** the main responsibility for reimbursement of cancer drugs is the specialist health care, linked to the Regional Health Authorities. The Decision Forum consisting of the CEOs of the four Regional Health Authorities make the decisions for introduction and reimbursement. The Norwegian Medicines Agency is responsible for setting the maximum prices on prescription medicines.  **MEAs/risk-sharing arrangements:** MEAs are not used in Norway due to administrative burden. | **Facilities collecting data for cancer care:** collected through prescription registers, national cancer registers, specific drug programs for both hospital and ambulatory medicines. For the former also through hospital records. Electronic patient charts are introduced or planned to be introduced in hospitals in all four health regions of Norway. Databases for sales statistics are also available. The National Cancer Registry dates back to the 1950s and contains a massive amount of data over time and across health care levels.  **Available for use and analysis by:** Health professionals, hospitals, reimbursement agencies/public services, Ministries of Health. Some data is publicly available. Manufacturers/industry do not have access to databases/sources within the healthcare system, but may request information/data from public national registries.  **Type of data:** aggregated data on diagnosis, indication, treatment duration, effectiveness, safety, patient reported outcome measures and expenditure. Different registries/sources exist and the categories of data recorded vary between them. Thus, some registries will not record all entities listed above, and they must be collected from different sources. The National Cancer Registry is the most complete aggregating data at a national level.  **Specific regulations for data access:** There are different conditions for access and use of data related to the different sources/databases/registries  **Data linkage between registers:** The National Cancer Registry collects data from the different levels of health care.  **Data robustness and validity:** data is well validated from the multiple database sources available and some with long history of data collection.  **Database update and analysis:** There are different routines for updates for different registries/databases up to the recent established electronic charts which collect data in real-time.  **Databases established since:** 1953 |
| Poland | **Separate procedure for ambulatory and hospital medicines:** No.  **Management level:** national for both ambulatory and hospital medicines.  **Key responsible authorities:** Ministry Of Health and National Health Fund.  **MEAs/risk-sharing arrangements:** nationally operating mechanism involve outcome schemes, confidential discounts, price: volume agreements, price cap agreements, conditional reimbursement.  **Challenges and opportunities with MEAs:** challenges with small patient populations. Opportunities - wider access to beneficial drugs. | **Facilities collecting data for cancer care:** data for ambulatory and inpatient cancer medicines is collected through national cancer registers and specific drug programs.  **Available for use and analysis by:** health professionals, reimbursement agencies/public services, Ministries of Health.  **Type of data:** individual-level and aggregated data. Data recorded for both ambulatory and inpatient medicines includes diagnosis, indication, treatment duration, effectiveness. Safety and expenditure data is also available for hospital medicines.  **Specific regulations for data access:** No  **Data linkage between registers:** there is possibility for registry linkage in hospital setting.  **Data robustness and validity:** problems of poor validity  **Database update and analysis:** N/A  **Databases established since:** 2015 |
| Romania | **Separate procedure for ambulatory and hospital medicines:** No.  **Management level:** national for both ambulatory and hospital medicines.  **Key responsible authorities:** the Ministry of Health, the National Agency of Medicine and Medical Devices (HTA Department), the National Health Insurance House and the Ministry of Finance (the reimbursement list update is approved through government decision).  **MEAs/risk-sharing arrangements:** operate nationally. Mainly involve price: volume agreements.  **Challenges and opportunities with MEAs:** increased patient access and decreased costs. however, long and bureaucratic process, not a genuine negotiation (fixed discounts) sometimes gaps between two consecutive agreements. | **Facilities collecting data for cancer care:** Ambulatory medicines data is collected through prescription registers, specific drug programs. For hospital medicines through hospital records, specific drug programs and regional cancer registers. Regional registers do not cover all pathologies. The National Program for oncology is mixed onco-haematology. Data for oncology medicines is collected separately from other DU data, because the funds are coming from the National Program of Oncology.  **Available for use and analysis by:** Data access and use is limited to owners which can include hospitals and health professionals or reimbursement agencies/public services.  **Type of data:** Both aggregated and individual-level data are collected including expenditure, diagnosis, indication, treatment duration and safety. However data is focused on consumption and expenditure, less on outcomes.  **Specific regulations for data access:** no.  **Data linkage between registers:** Data can be linked across registers but mainly focused on expenditure, and access limited to data owners (NHIH).  **Data robustness and validity:** Issues with data validation as lack of human resources.  **Database update and analysis:** There are a number of regional registries but only two function properly, and data is not properly filled for the others, thus database update is inconsistent across the registers. However data tends be analyzed quarterly by the NHIH and extrapolated for the country based on the two main functional registers.  **Databases established since:** N/A |
| Scotland (United Kingdom) | **Separate procedure for ambulatory and hospital medicines:** Yes.  **Management level:** nationally for both ambulatory and hospital medicines.  **Key responsible authorities:** Scottish Medicines Consortium. Local health boards may also make decisions on individual cases for orphan or very high cost medicines.  **MEAs/risk-sharing arrangements:** in operation nationally, mainly confidential discounts but different MEA with uptake varying across regions in Scotland, depending on likely patient numbers/cost savings as they can be very time consuming to set up. | **Facilities collecting data for cancer care:** hospital records, and national cancer registers record data for hospital and ambulatory medicines; for the latter also through prescription registers. Dispensed prescription for ambulatory medicines are collected by the Prescribing Information System (PIS) to ensure community pharmacies are reimbursed. Some hospitals have electronic prescribing and so these prescriptions will also be recorded. The national cancer registry should record whether or not patients have anti-cancer treatment for their primary but may not always identify medicines given for metastatic disease or recurrence.  **Available for use and analysis by:** Health professionals, hospitals, reimbursement agencies/public services, Ministries of Health. Public and academia could apply to access some of this data in an anonymized way.  **Type of data:**  aggregated data, expenditure, treatment duration, safety data. Treatment duration could be calculated generally. Indication may be captured depending on the prescribing set-up.  **Specific regulations for data access:** Data access regulations vary depending on how many health boards are involved and on the intention for accessing the data i.e. research or audit.  **Data linkage between registers:** linkage is possible  **Data robustness and validity:** some limitations  **Database update and analysis:** N/A  **Databases established since:** 1958 |
| Slovakia | **Separate procedure for ambulatory and hospital medicines:** Yes  **Management level:** nationally for ambulatory medicines, both nationally and regionally for hospital medicines.  **Key responsible authorities:** Nationally, the Ministry of Health, regionally (negotiations for hospital drugs) no specific regional authorities.  **MEAs/risk-sharing arrangements:** in operation nationally, including confidential discounts, price cap agreements. MEAs rarely used but more regulations being put in place. | **Facilities collecting data for cancer care:** prescription registers (health insurance data collected in the central database of the National Institute of Health Care Information - NCZI) and national cancer registers (National Oncological Registry - NOR). For hospital medicines also through hospital records.  **Available for use and analysis by:** Publicly available. More detailed data available upon request.  **Type of data:** aggregated data on diagnosis and expenditure mainly. NCZI collects hospital inpatient care data - drugs, costs, prices, packages, diagnosis. NOR (part of NCZI) collects epidemiological data on incidence and mortality.  **Specific regulations for data access:** Some aggregated data is publicly available at NOR and NCZI. Some "big data" - not specific for oncology but including - available at MoH for scientific reasons.  **Data linkage between registers:** no linkage possibility. Aggregated data at NCZI (NOR) without linkage. Some data available after a specific request. Data in HIC most robust with linkage.  **Data robustness and validity:** do not know  **Database update and analysis:** generally annual update but data for NOR data is available up to 2012 (delay). Quarterly for NCZI data from HIC.  **Databases established since:** 1978 |
| Slovenia | **Separate procedure for ambulatory and hospital medicines:** No  **Management level:** nationally for ambulatory and hospital medicines.  **Key responsible authorities:** The Agency for Medicines and National Health Insurance.  **MEAs/risk-sharing arrangements:** in operation nationally. Include confidential discounts, price: volume agreements, price cap agreements, pay-back schemes.  **Challenges and opportunities with MEAs:** High prices, high expenditures, long-term use for more patients. | **Facilities collecting data for cancer care:** mainly through prescription registers for ambulatory care drugs and through hospital records for inpatient medicines.  **Available for use and analysis by:** hospital and reimbursement agencies/public services.  **Type of data:** Individual-level and aggregated data is collected. Expenditure and treatment duration data is recorded for both ambulatory and hospital medicines, and indication is also available for the latter.  **Specific regulations for data access:** strict regulations for healthcare data access  **Data linkage between registers:** datasets are separate and no linkage is available.  **Data robustness and validity:** Data is well validated  **Database update and analysis:** regular weekly updates and analysis conducted monthly and annually.  **Databases established since:** 2013. |
| Catalonia (Spain) | **Separate procedure for ambulatory and hospital medicines:** Yes  **Management level:** regional and national for ambulatory medicines, and mainly national for hospital medicines.  **Key responsible authorities:** Ministry of Health (national), Department/Service of Health (regionally). Medicine prices and financial conditions are fixed by the Ministry of Health as the maximum reimbursed price. However, regions maintain their autonomy with regards to operative aspects of healthcare provision, including the efficient incorporation of medicines across their territories (reimbursement procedures). The management of reimbursement and invoicing of medicines is one of the functions of the Catalan Heath Service in Catalonia.  **MEAs/risk-sharing arrangements:** operating nationally and regionally. Include outcome schemes, confidential discounts, price: volume agreements, price cap agreements.  **Challenges and opportunities with MEAs:** Opportunities – Incorporation of drug innovation for patients under optimal conditions, reduce uncertainties on clinical outcomes or budget and economic impact. Challenges – Cost in terms of staff time in order to follow up and access the conditions. | **Facilities collecting data for cancer care:** for ambulatory care medicines mainly through specific drug program/registers (orphan drugs, immunosuppressant drugs, and drugs for HIV and HCV infections).  **Available for use and analysis by:** Health professionals, hospitals, reimbursement agencies/public services.  **Type of data:** individual-level data on diagnosis, indication, treatment duration, effectiveness and safety, dispensing. Expenditure data is informed through health service invoicing.  **Specific regulations for data access:** Only for management and clinical applications for health system professionals and according to EU regulation on the protection of persons with regard to the processing of personal data and on the free movement of such data. For public research purposes, the information of the Patient and Treatment Registry is available through Catalan Agency for Health Quality and Evaluation (AQuAS).  **Data linkage between registers:** data can be linked between databases in ambulatory and hospital setting.  **Data robustness and validity:** some limitations - Patient and Treatment Registry data validation concluded that 85% of the items checked were traceable in clinical records. Integral Plan of Quality is on the way to document this.  **Database update and analysis:** the data is analyzed continuously for different information needs for management and clinical applications (study drugs use, health outcomes, quality indicators based on effectiveness, to manage risk sharing agreement and for budgetary projections, amongst other uses). Some reviews are programed annually.  **Databases established since:** 2012 |
| Sweden | **Separate procedure for ambulatory and hospital medicines:** Yes  **Management level:** nationally for ambulatory medicines and both national and regional management for hospital medicines.  **Key responsible authorities:** at the national level, the national Reimbursement Agency (Dental and Pharmaceutical Benefits Agency, TLV). Each region responsible for the budget, tenders and recommendations in the region. There are 21 independent region in Sweden jointly working together for tertiary care in 6 health regions. The Swedish Association of Local Authorities and Regions (SALAR) and the New Therapies (NT) Council, a group of experts that supports the Swedish county councils on questions concerning new drug therapies, with the aim of enabling equal drug treatment for patients throughout the country.  **MEAs/risk-sharing arrangements**: in operation at national and regional level. Include  outcome schemes, confidential discounts, price cap agreements.  **Challenges and opportunities with MEAs:** The main challenge is the many stakeholders involved, with the national reimbursement agency operating under the state responsible for price and reimbursement in ambulatory care, and the regions having the budget for hospital care. Some regions are very small, having difficulties to manage new medicines. The opportunities is the coordinated work led by the New medicines council and the Swedish tradition with large registers and epidemiologic competence. New national legislation may come. There is need to collect RWD. | **Facilities collecting data for cancer care:** prescription register, hospital records, specific drug programs, regional and national cancer registers collect data for ambulatory and hospital medicines. There is also a national platform for collecting clinical data on cancer - INCA, run by regional cancer centers in collaboration. It is not yet complete. There is a special national network of regional cancer centers keeping registers and developing guidelines. There is also a specific national cancer register from 1958 held by The National Board of Health and Welfare. It does not contain data on medicines, but it can be used in record-linkage.  **Available for use and analysis by:** health professionals, hospital use, reimbursement agencies/public services, Ministries of health. Can also be used for research after ethical approval.  **Type of data:** both aggregated and individual-level data is collected on diagnosis, treatment duration, indication, safety and expenditure. This varies a lot between registers, regions and cancer forms. Such data is the most available on a national level. There is also registration of other types of data in some registers.  **Specific regulations for data access:** National legislation on health registries and patient data. Very limited direct access to data outside the clinical/hospital patients are treated, but most data are available for research after ethical approval.  **Data linkage between registers:** Almost all databases in Sweden use the same patient identifier - the personal id-number. It is rather easy at the national level with two national agencies linking data for research and certain analyses, but hospital medical records data are spread in different systems. They can be linked in theory, but requires a lot of permissions.  **Data robustness and validity**: there are significant issues with data validity. Data is not automatically transferred but there is manual reporting into the registry from patient charts, and there is low reporting rate. It is difficult to check validity as there are different reporting criteria, and not all volumes of cancer drugs are covered.  **Database update and analysis:** Ambulatory drugs are registered continuously, but there is very limited data on hospital drugs. No regular updates and analysis, which can be carried out monthly/annually.  **Databases established since:** Cancer registry was established since 1958, and pharmaceutical treatments started being included in the databases in the 2000s |
