# Supplementary material for: Challenges and Opportunities With Routinely Collected Data on the Utilization of Cancer Medicines. Perspectives From Health Authority Personnel Across 18 European Countries
Source: Front Pharmacol. 2022 Jun 16;13:873556. doi: 10.3389/fphar.2022.873556 (PMC9295616; doi:10.3389/fphar.2022.873556)
Supplement: Supplementary file 2 [file Table2.docx]

Supplementary File 3

**Supplementary table 2.** Key oncology medicines mentioned by respondents in the questionnaire.

| **Medicines mentioned for one country** | **Medicines mentioned for 2-3 countries** | **Medicines mentioned for multiple countries** |
| --- | --- | --- |
| 5-fluorouracil, Abemaciclib, Acalabrutinib, Alectinib, Alemtuzumab, Apalutamid, Atezolizumab, Brigatinib, Carboplatin, Cetuximab, Cisplatin, Cladribine, Cobimetinib, Crizotinib, Dakomatinib, Durvalumab, Encorafenib, Gemcitabinum, Imatinib, Ipilimumab, Ixazomib, Lenvatinib, Leucovorin, Lorlatinib, Megestrol, Methotrexatum, Mistle toe, Nilotinib, Olaparib, Onivyde pegylated liposomal, Osimertinib, Pertuzumab, Ribociclib, Rituximab, Sunitinib, Tamoxifen, Tisagenlecleucel, Triptorelinum | Abiraterone, Avelumab, Bevacizumab,  Daratumumab Enzalutamid Hydroxycarbamide, Lenalidomid, Oomalidomid, Ruxolitinib, Trastuzumab emtansine | Ibrutinib, Nivolumab, Paclitaxel, Palbociclib, Pembrolizumab, Trastuzumab |
