# Supplementary material for: Challenges and Opportunities With Routinely Collected Data on the Utilization of Cancer Medicines. Perspectives From Health Authority Personnel Across 18 European Countries
Source: Front Pharmacol. 2022 Jun 16;13:873556. doi: 10.3389/fphar.2022.873556 (PMC9295616; doi:10.3389/fphar.2022.873556)
Supplement: Supplementary file 3 [file DataSheet1.pdf]

frontiers

Supplementary File 1

Questionnaire outline.

Questionnaire on the availability of patient-level datasets for oncology across Europe

---

Background and research purpose

Global medicine expenditure has been increasing in recent years, and a large share is attributed to increased development and launch of new high-priced medicines for complex diseases including cancer, exacerbated by unmet medical need and the emotive nature of these diseases. This has driven concern across Europe and elsewhere that the increasing costs of oncology medicines will become unsustainable for health systems and for universal healthcare access if this trend continues. Early market access of new oncology medicines often lacking robust clinical data generates uncertainty over their value and likely cost-effectiveness in routine clinical care further compounding the situation. Better understanding of the availability of patient-level datasets among different European countries could provide opportunities for improving data robustness and decision-making, particularly to address issues with pricing and affordability of new cancer medicines balancing the unmet need for new cancer drugs. This is particularly important with new cancer medicines being launched in small targeted populations often with limited data and high prices.

The aim of this survey is to collect information on the availability of drug utilization and expenditure data on newly launched cancer medicines in different European countries, particularly concerning the availability of patient-level datasets that can help evaluate the utilization, effectiveness and safety of these new medicines in routine clinical care. Additionally, to highlight different country perspectives on important policy questions, as well as possibilities and challenges for collaboration across Europe and how this could help with future developments and pricing models. The research conducted is part of a master thesis for the Global Health programme at the Karolinska Institutet.

Informed consent

Your participation in this survey is voluntary. You may refuse to take part in the research or exit the survey at any time without penalty. You are free to decline to answer any particular question you do not wish to answer for any reason.

At the end of the survey you will be asked if you are interested in participating in an additional interview. If you choose to provide contact information, your survey responses may no longer be anonymous to the researcher. However, no identifying information would be included in any publications or presentations based on these data, and your responses to this survey will remain confidential.

☐ I agree

Contact Information

First Name

Last Name

Employer/Organization

Job title/role

*Country

*City/Region

Phone

Email

General - Availability of cancer medicines

What cancer medicines, both in ambulatory and inpatient care, are currently the major focus in your country in terms of patient use and budget? Why is this? (Indicate up to 5 medicines, their class and therapeutic indication).

Pricing and Reimbursement

Are there separate pricing and reimbursement procedures for new oncology medicines for use exclusively in hospitals (in-patient care) versus ambulatory medicines (includes all medicines administered in outpatients)?

- ◯ Yes
- ◯ No

Is pricing and reimbursement for new oncology medicines regulated nationally or regionally?

Ambulatory medicines

- ◯ Nationally
- ◯ Regionally
- ◯ Both

Hospital medicines

- ◯ Nationally
- ◯ Regionally
- ◯ Both

What are the key authorities involved in pricing and reimbursement decisions both nationally and regionally (if pertinent)?

Are there managed entry agreements (MEAs)/schemes or risk-sharing arrangements in operation either nationally or regionally in your countries to enhance the affordability of new cancer medicines?

National

- ◯ Yes, 5 or more
- ◯ Yes, less than 5
- ◯ No

Regional

- ◯ Yes, 5 or more
- ◯ Yes, less than 5
- ◯ No

What type of MEAs/schemes do you have? (Select all that apply)

- ☐ Outcome schemes
- ☐ Confidential discounts
- ☐ Price: volume agreements
- ☐ Price cap agreements
- ☐ Other (Please specify)

What are some opportunities and challenges of these arrangements especially for new cancer medicines in your country context?

Databases for drug utilization and patient-level data

What facilities or settings collect drug utilization data for cancer care in your country including any MEAs? (Select all that apply)

Ambulatory care

- [ ] Hospital records
- [ ] Prescription registers
- [ ] Specific drug programs/registers
- [ ] Regional cancer registers
- [ ] National cancer registers
- [ ] Other

Hospital care

- [ ] Hospital records
- [ ] Prescription registers
- [ ] Specific drug programs/registers
- [ ] Regional cancer registers
- [ ] National cancer registers
- [ ] Other

If "other" please specify:

Do they differ from structures collecting other types of drug utilization data for other therapeutic areas?

- ◯ Yes
- ◯ No

If yes please elaborate:

[ ]

For which entities these databases/registries are available for analysis and use? (select all that apply)

- ☐ Health professionals (clinicians, nurses, pharmacologists, pharmacists)
- ☐ Hospital use
- ☐ National or regional reimbursement agencies/public services
- ☐ Ministries of Health
- ☐ Publicly available
- ☐ Manufacturers/industry
- ☐ Other (Please specify)

[ ]

Do you collect individual level data or aggregated data for individual medicines?

- ◯ Individual-level data
- ◯ Aggregated data
- ◯ Both

What type of utilization data is recorded? (select all that apply)

Ambulatory care

- [ ] Diagnosis
- [ ] Indication
- [ ] Treatment duration
- [ ] Effectiveness (survival, PFS, quality of life, other)
- [ ] Safety (adverse events)
- [ ] Patient Reported Outcome Measures (PROMs)
- [ ] Expenditure (resource utilization)
- [ ] Dispensing data
- [ ] Other

Hospital care

- [ ] Diagnosis
- [ ] Indication
- [ ] Treatment duration
- [ ] Effectiveness (survival, PFS, quality of life, other)
- [ ] Safety (adverse events)
- [ ] Patient Reported Outcome Measures (PROMs)
- [ ] Expenditure (resource utilization)
- [ ] Dispensing data
- [ ] Other

If "other" please specify:

Are there specific regulations for data access and sharing?

○ Yes ○ No

If yes please elaborate:

Is there possibility for data linkage between registries/health records in ambulatory and hospital settings?

Ambulatory care

○ Yes

○ No

Hospital care

○ Yes

○ No

Please elaborate:

Is the data collected/available robust and valid?

○ Yes, but some limitations ○ Yes, well validated ○ No, problems of poor validity

○ Do not know

Please elaborate:

How long have databases for cancer patients been in place in your country?

MM DD YYYY

How often are databases updated and how often is data analysed?

Updated

- [ ] Weekly
- [ ] Monthly
- [ ] Annually
- [ ] Other

Analysed

- [ ] Weekly
- [ ] Monthly
- [ ] Annually
- [ ] Other

If "other" please specify:

What are the advantages and disadvantages of data collection systems in your county, and what are barriers to collect data and establish registries for safety and outcomes of new cancer medicines across the sectors?

Future improvements and developments

Outline any suggestions to improve data collection in your country in the different care sectors:

Outline barriers or possibilities for cross-national collaboration and data-linkage across European countries to more rapidly ascertain real-life experience. How could barriers be overcome?

Concluding remarks

Are you aware of relevant publications about this that you could provide as supporting material either for your country or other European countries?

Can you suggest additional contacts that could help with this questionnaire?

Do you agree to be contacted for a potential additional interview by phone, email or zoom?

- ◯ Yes
- ◯ No
