# Supplementary material for: Challenges and Opportunities With Routinely Collected Data on the Utilization of Cancer Medicines. Perspectives From Health Authority Personnel Across 18 European Countries
Source: Front Pharmacol. 2022 Jun 16;13:873556. doi: 10.3389/fphar.2022.873556 (PMC9295616; doi:10.3389/fphar.2022.873556)
Supplement: Supplementary file 4 [file Table3.DOCX]

Supplementary File 4

**Pricing and reimbursement management and funding mechanisms, PDF format (.pdf)**

1. Pricing and reimbursement management at national or regional level
2. Use of managed entry agreements (MEAs) or other risk-sharing arrangements at national or regional level
3. Types of MEAs or other schemes used
